# Monitoring the Efficacy of Tafamidis in ATTR Cardiac Amyloidosis by MRI-ECV: A Systematic Review and Meta-Analysis

**DOI:** 10.3390/tomography10080097

**Published:** 2024-08-16

**Authors:** Shingo Kato, Mai Azuma, Nobuyuki Horita, Daisuke Utsunomiya

**Affiliations:** 1 Department of Diagnostic Radiology, Graduate School of Medicine, Yokohama City University, 3-9, Fukuura, Kanazawa-ku, Yokohama 236-0004, Kanagawa, Japan; shingo.m12226@gmail.com; 2 Department of Cardiology, Kanagawa Cardiovascular and Respiratory Center, 6-16-1 Tomioka-Higashi, Kanazawa-ku, Yokohama 236-0051, Kanagawa, Japan; mai.smile.mai2@gmail.com; 3 Chemotherapy Center, Yokohama City University Hospital, 3-9, Fukuura, Kanazawa-ku, Yokohama 236-0004, Kanagawa, Japan; horitano@yokohama-cu.ac.jp

**Keywords:** cardiac amyloidosis, cardiac MRI, extracellular volume fraction, tafamidis, meta-analysis

## Abstract

Background: The usefulness of monitoring treatment effect of tafamidis using magnetic resonance imaging (MRI) extracellular volume fraction (ECV) has been reported. Objective: we conducted a meta-analysis to evaluate the usefulness of this method. Methods: Data from 246 ATTR-CMs from six studies were extracted and included in the analysis. An inverse variance meta-analysis using a random effects model was performed to evaluate the change in MRI-ECV before and after tafamidis treatment. The analysis was also performed by classifying the patients into ATTR-CM types (wild-type or hereditary). Results: ECV change before and after tafamidis treatment was 0.33% (95% CI: −1.83–2.49, I^2^ = 0%, p = 0.76 for heterogeneity) in the treatment group and 4.23% (95% CI: 0.44–8.02, I^2^ = 0%, p = 0.18 for heterogeneity) in the non-treatment group. The change in ECV before and after treatment was not significant in the treated group (p = 0.76), but there was a significant increase in the non-treated group (p = 0.03). There was no difference in the change in ECV between wild-type (95% CI: −2.65–3.40) and hereditary-type (95% CI: −9.28–4.28) (p = 0.45). Conclusions: The results of this meta-analysis suggest that MRI-ECV measurement is a useful imaging method for noninvasively evaluating the efficacy of tafamidis treatment for ATTR-CM.

## 1. Introduction

Transthyretin Amyloid Cardiomyopathy (ATTR-CM) is a progressive and fatal disease caused by the accumulation and extracellular deposition of insoluble amyloid fibers derived from transthyretin in the myocardium [1,2]. Traditionally considered a rare disease with a poor prognosis, recent epidemiological data indicate that the incidence of cardiac amyloidosis has increased over the past decade, with wild-type ATTR-CM being a major contributing factor [3]. It is also reported to be present in heart failure with preserved ejection fraction [4] and aortic stenosis [5], and is by no means rare. Tafamidis was approved as a breakthrough treatment for ATTR-CM and significantly improved mortality in patients with ATTR-CM compared to placebo [6]. However, because tafamidis stabilizes and inhibits disease progression rather than curing it [7], its effects are not readily apparent to clinicians or patients. Therefore, monitoring of treatment efficacy is clinically important.

Non-invasive assessment of extracellular volume fraction (ECV) by cardiac MRI is useful in the diagnosis of cardiac amyloidosis [8,9,10]. A significant increase in MRI-ECV is known to be present in cardiac amyloidosis, with reported diagnostic sensitivity and specificity of 89% [11]. Consequently, both ACC/AHA and ESC clinical guidelines have proposed diagnostic algorithms for cardiac amyloidosis using cardiac MRI [12,13]. Recent reports also suggest that ECV in ATTR cardiac amyloidosis increases over time, but in patients treated with tafamidis, ECV remains stable during follow-up, indicating disease progression inhibition [14,15,16]. Thus, while small single-center studies have reported the utility of MRI-ECV in monitoring the therapeutic effect of tafamidis, comprehensive systematic reviews and meta-analyses on this topic are lacking. The aim of this study is to conduct a meta-analysis to assess the utility of MRI-ECV in monitoring the therapeutic effects of tafamidis treatment.

## 2. Materials and Methods

### 2.1. Search Strategy and Selection Criteria

We followed the methodology proposed by the Cochrane Collaboration and adhered to the reporting criteria outlined in the Preferred Reporting Items for Systematic Review and Meta-analysis (PRISMA) guidelines for the year 2020 [17]. On 5 February 2024, a comprehensive database search was conducted using PubMed, Web of Science Core Collection, Cochrane advanced search, and the EMBASE electronic database. The following keywords were employed: “tafamidis”, “extracellular volume” “ECV”, “MRI”, and “ATTR-CM” etc. (Supplementary File S1). Eligibility criteria encompassed all articles that presented pre- and post-treatment MRI-ECV values of tafamidis, which were discoverable through the specified keywords. Exclusion criteria were limited to case reports, animal studies, and non-English articles. Two reviewers (SK and MA) screened all titles and abstracts in the search results, and potentially relevant studies were subjected to a full review for eligibility. Disagreements were resolved by a third reviewer. The study protocol was registered with the University Medical Information Network (registration number: UMIN000053556). Institutional review board approval was not deemed necessary since this study constituted a meta-analysis and did not involve the use of clinical patient information. Data were also extracted from the literature concerning changes in MRI-ECV over time in the control group (ATTR-CM without Tafamidis therapy).

### 2.2. Outcome Assessment

The primary outcome measure in this meta-analysis is the change in MRI-ECV before and after the treatment of ATTR-CM, with and without tafamidis. Two reviewers (SK and MA) extracted the following study characteristics for both the control and heart disease groups: author names, publication year, country of origin, patient disease, age, sex, and MRI parameters, including MRI-ECV values.

Specifically, we extracted MRI-ECV values before Tafamidis administration and conducted a meta-analysis of the mean differences with post-administration MRI-ECV values. Additionally, to elucidate the differences between wild-type ATTR-CM and hereditary ATTR-CM, a stratified analysis was performed, and a meta-analysis of the mean differences in MRI-ECV values before and after treatment for each type was conducted. These mean differences were defined as the primary outcome of this study.

### 2.3. Assessment of Risk of Bias

To assess risk of bias, the Newcastle-Ottawa Quality Assessment Scale and case-control studies were used [18]. To assess publication bias, funnel plots were shown for ECV values before and after tafamids treatment in ATTR-CM patients.

### 2.4. Data Integration and Statistical Analysis

Meta-analysis was conducted using RevMan 5.41 (Cochrane Collaboration, London, UK). Pooled MRI-ECV values were estimated using an inverse variance method with a random effects model. The chi-square test was employed to compare MRI-ECV data between the tafamidis-treated and non-tafamidis-treated groups. Heterogeneity was assessed using the I^2^ statistic, where 0% indicates no heterogeneity, and 100% implies strong heterogeneity [19]. To examine publication bias, Begg’s test was employed, and Kendall’s tau was calculated. A p-value < 0.10 for Begg’s test was considered statistically significant, while for other statistical methods, a p-value <0.05 was considered statistically significant.

## 3. Results

### 3.1. Characteristics of Included Studies

**Figure 1:**
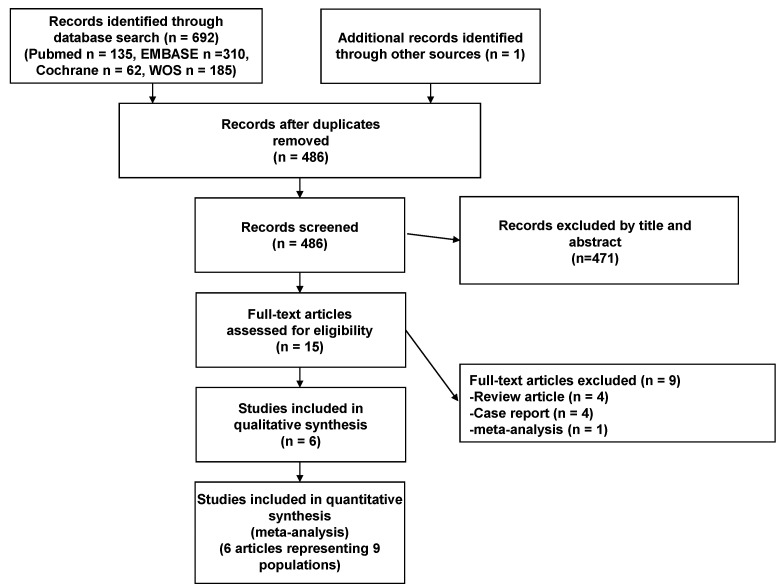
PRISMA flow diagram.

Six eligible papers [14,15,16,20,21,22] were finally selected from 486 candidate papers (Figure 1), and the characteristics of the included studies are summarized in Table 1. Data from 246 ATTR-CMs were included in the analysis. Patient age and sex are summarized in Table 1. The publication years ranged from 2021 to 2024. Two studies were published from Germany [15,22], the UK [20], Austria [14], Japan [16], and Taiwan [21], one each. Four studies were retrospective single-center studies [15,16,20,22] and two were retrospective analyses of prospective cohorts [14,21]. Five studies used a 1.5T MRI system [14,15,20,21,22], and in one study, the magnetic field strength was unknown [16]. Two studies reported ECV data for tafamidis-treated and non-tafamidis-treated patients [14,15], while one study compared Patisiran-treated and non-tafamidis-treated patients, so only ECV data for the control group were extracted [20]. Three studies provided ECV data for the tafamidis-treated group only [16,21,22]. A total of nine cohorts of data were analyzed: four with Tafamidis 61 mg [14,15,21,22], one with Tafamidis 20 mg [14], one with Tafamidis dose unknown [16], and three with Tafamidis not administered [14,15,20] (Table 2). 

### 3.2. MRI-ECV Changes in Tafamidis-Treated and Non-Treated ATTR-CM Patients

**Figure 2:**
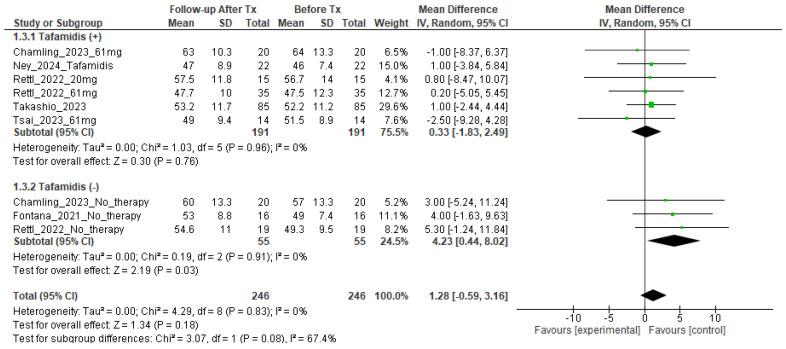
Comparison of the change in MRI-ECV before and after tafamidis treatment.

**Figure 3:**
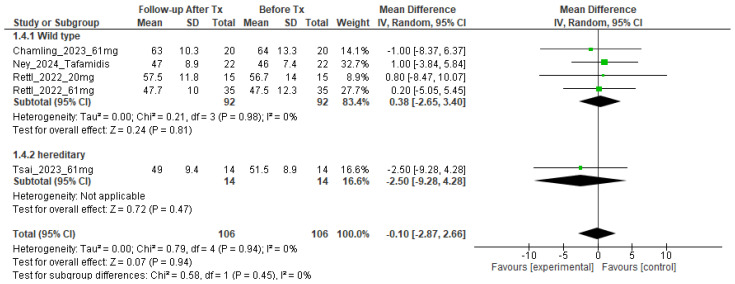
Comparison of the change in MRI-ECV before and after tafamidis treatment of wild type and hereditary ATTR-CM.

The MRI-ECV data for each study are summarized in Table 2. Left ventricular ECV values before tafamidis treatment were 52.4% (95% confidence interval (CI): 48.1–56.8%, I^2^ = 86%, p < 0.001 for heterogeneity) in the treated group and 51.2% (95% CI: 46.8–55.6%, I^2^ = 81%, p < 0.001 for heterogeneity) in the non-treated group. Left ventricular ECV after tafamidis treatment was 52.7% (95% CI: 48.1–57.2%, I^2^ = 88%, p < 0.001 for heterogeneity) in the treatment group and 55.4% (95% CI: 51.5–59.3%, I^2^ = 84%, p < 0.001 for heterogeneity) in the non-treatment group. The change in ECV was 0.33% (95% CI: −1.83–2.49, I^2^ = 0%, p = 0.76 for heterogeneity) in the treatment group and 4.23% (95% CI: 0.44–8.02, I^2^ = 0%, p = 0.18 for heterogeneity) in the non-treatment group (Figure 2). The change in ECV before and after treatment was not significant in the treated group (p = 0.76), but there was a significant increase in the non-treated group (p = 0.03). We also compared the change in ECV for Tafamidis, classified as wild type and herrdirary ATTR-CM: 0.38% (95% CI: −2.65–3.40, I^2^ = 0%, p = 0.81 for heterogeneity) in wild type versus 0.38% in hereditary −2.50 (95%CI: −9.28–4.28), and there was no difference in the change in ECV between the two groups (p = 0.45) (Figure 3). The quality ratings of the studies for the assessment of risk of bias are summarized in Table S1. Overall, 6 of 6 studies (100%) were rated as high quality (scoring more than 80% on the quality scales). The Begg’s test detected a possible publication bias for the measurement of MRI-ECV in ATTR-CM before tafamidis therapy (Kendall’s tau = 0.5353, p = 0.046). However, no significant publication bias was detected for MRI-ECV measurement in ATTR-CM after tafamidis therapy (Kendall’s tau = 0.3889, p = 0.18) (Figures S1 and S2).

## 4. Discussion

The results of our meta-analysis suggest that MRI-based assessment of ECV may be useful in monitoring the effects of tafamidis in ATTR-CM. Since tafamidis is a drug that suppresses the deposition and progression of amyloid fibrils, the effect of treatment is difficult to assess clinically, and MRI-ECV, which can monitor the effect of treatment, may play a significant role.

Early diagnosis and duration length of the disease is important because tafamidis is an important drug that improves prognosis in patients with early-stage ATTR-CM, but is not effective in patients with severe ATTR-CM [6]. Monitoring of disease progression is also essential to identify nonresponders, especially in terms of significant treatment costs [23]. However, monitoring drug efficacy is not easy because tafamidis is a drug that slows disease progression and does not improve symptoms or markedly improve blood laboratory data. Several studies have been conducted on monitoring the efficacy of tafamidis using noninvasive imaging [24,25,26]. A recently published meta-analysis analyzed pre- and post-treatment changes in echocardiographic left ventricular ejection fraction, ventricular septal thickness (IVS), and global longitudinal strain [27]. This paper reported that left ventricular ejection fraction decreased slightly after tafamidis treatment, but IVS thickness and global longitudinal strain did not change before or after treatment, which could be used to determine the effect of treatment [27]. However, echocardiography has some problems: it is affected by the patient’s physique, the image quality is highly dependent on the operator, and the reproducibility of the measurement is somewhat problematic. MRI, on the other hand, overcomes these limitations of echocardiography and is the gold standard imaging method for characterizing myocardial tissue [28]. To the best of our knowledge, our meta-analysis is the first to analyze MRI-ECV changes due to tafamidis in patients with ATTR-CM. Of great interest was the low heterogeneity in the meta-analysis of MRI-ECV change: 0.33% of the treatment group had I^2^ = 0% heterogeneity in MRI-ECV change, despite greater variability in baseline MRI-ECV in the tafamidis treatment group (p = 0.76). I^2^ = 0% heterogeneity compared to 4.23% in the non-treated group (p = 0.18 heterogeneity). These results may suggest that Tafamidis MRI-ECV monitoring is a reliable indicator of ATTR-CM in myocardial injury of varying severity.

The difference in responsiveness of wild-type ATTR-CM and hereditary ATTR-CM to tafamidis is also of interest. Since a previous study have shown no prognostic difference between wild-type ATTR-CM and hereditary ATTR-CM treated with tafamids [27], we also compared the ECV changes in the two groups and found no significant difference between the two groups (Figure 3). Our results were consistent with that of previous study. Also, patisiran, a TTR-specific small interfering RNA (siRNA), is also known to be effective for hereditary ATTR-CM [29]. Fontana et al. found that patisiran treatment was associated with a lower ECV (adjusted mean difference between groups: 6.2% [95% CI]: 9.5% to 3.0%) [20]. Furthermore, ATTR antibodies have recently been developed and reported to produce significant improvements in MRI-ECV [30]. Considering these lines of evidence, MRI-ECV may be useful for monitoring not only tafamidis but also drugs that improve amyloid deposition, such as patisiran and ATTR antibodies. Although evidence is limited at this time, evaluation of myocardial ECV may suggest optimal treatment and monitoring of patients with ATTR-CM and may contribute to treatment decisions and improve prognosis.

Cardiac MRI plays a crucial role in the diagnosis of cardiac amyloidosis. Specifically, LGE MRI detects abnormal gadolinium uptake in the myocardium, indicative of amyloid deposition with expanded interstitium, demonstrating subendocardial enhancement [31]. When LGE becomes transmural, it reflects disease progression and is a sign of poor prognosis [32]. Additionally, the diagnostic capability of myocardial ECV is high, with a sensitivity of 89% and specificity of 89%, as reported in a meta-analysis [11]. However, it is currently challenging to definitively differentiate between ATTR and AL using MRI alone. Nuclear medicine imaging, such as pyrophosphate scintigraphy, is useful for distinguishing between the two types [13]. The effect of Tafamidis on MRI-ECV is likely due to its main mechanism of preventing amyloid deposition and inhibiting the progression of interstitial expansion. There are also studies evaluating the impact of Tafamidis on MRI parameters other than ECV. In the group treated with Tafamidis, there was no decrease in LVEF, increase in LV mass, or increase in native T1 one year after the start of treatment compared to the non-treated cardiac amyloidosis group [15]. MRI parameters other than ECV may also be useful for monitoring the effects of Tafamidis.

This study has several limitations. First, this meta-analysis needs to investigate the utility of MRI-ECV in larger prospective intervention studies because the studies included in the analysis are predominantly small numbers of backward-looking studies. Second, MRI-ECV cannot be evaluated in patients with renal dysfunction because it requires gadolinium contrast administration; since ATTR-CM is also complicated by renal dysfunction, echocardiography and myocardial scintigraphy may play an important role in these patients. Recently, the utility of ECV with computed tomography (CT) has also been demonstrated, and CT may also be an alternative to MRI in patients with severe renal dysfunction [33,34,35]. Third, MRI native T1 time data were also provided, but meta-analysis could not be performed because the reference values varied widely from institution to institution. Forth, the number of patients included in the studies is very small, limiting comprehensive evaluations. Additionally, this meta-analysis has several other limitations. Non-English papers and research were not considered, which could introduce language bias. Most of the included studies are retrospective, presenting a significant limitation due to potential biases that may affect the final results. Lastly, it is challenging to define the impact of ECV in non-ATTR patients with amyloidosis, making it difficult to compare results with other conditions. Fifth, the study focuses solely on a meta-analysis of papers measuring ECV by MRI in tafamidis-treated patients versus controls with amyloidosis, and the results are inconclusive, indicating that MRI could be useful in this setting. Additionally, the timing of treatment initiation and the duration of the disease were not consistently reported in the analyzed literature, making it difficult to compare early and late diagnoses.

In conclusion, our meta-analysis highlights the potential utility of MRI-based assessment of ECV in monitoring tafamidis effects on ATTR-CM, given its challenge in clinical evaluation. MRI-ECV is a more reliable indicator for assessing ATTR-CM and may improve treatment decisions and outcomes. More research is needed to fully establish its role in guiding therapeutic strategies.

## Figures and Tables

**Table 1:** Characteristics of included studies.

1st Author	Study Design	Treatment	Age, Years	Sex, Male	Number of ATTR-CM	Type of Amyloidosis
Fontana et al. [20]	Retrospective single center	No therapy	69 (62 to 80)	88%	16	Wild type and hereditary
Rettl et al. [14]	Retrospective analysis of registry data	No therapy	78.4 (5.8)	78.9%	19	Wild type
		Tafamidis 20 mg	74.9 (3.7)	86.7%	15	
		Tafamidis 61 mg	78.3 (6.3)	82.9%	35	
Chamling et al. [15]	Retrospective single center	No therapy	80 (75–82)	75%	20	Wild type
		Tafamidis 61 mg	76 (73–81)	90%	20	
Takashio et al. [16]	Retrospective single center	Tafamidis	75.6 ± 5.3	88%	85	Wild type and hereditary
Tsai et al. [21]	Retrospective analysis of prospective cohort	Tafamidis 61 mg	62.1 ± 4.9	93%	14	hereditary
Ney et al. [22]	Retrospective single center	Tafamidis 61 mg	79.0 ± 6.4	88%	22	Wild type

ATTR-CM, Transthyretin Amyloid Cardiomyopathy. Values are mean ± standard deviation or median (inter quartile range).

**Table 2:** Summary of changes in native T1 times and ECV by tafamidis therapy.

1st Author	Follow-Up Duration	Treatment	Magnetic Field Strength	Measurement Site	Native T1 Baseline, Msec	Native T1 Follow-Up, Msec	ECV Baseline, %	ECV Follow-Up, %
Fontana et al. [20]	1 year	No therapy	1.5 T	global	1124 (1091–1172)	1130 (1110–1167)	49 (41–51)	53 (46–58)
Rettl et al. [14]	1 year	No therapy	1.5 T	global	1097 ± 43.5	1100.7 ± 43.4	49.3 ± 9.5	54.6 ± 11.0
		Tafamidis 20 mg		global	1135 ± 105	1127 ± 44	56.7 ± 14.4	57.5 ± 11.8
		Tafamidis 61 mg		global	1111.1 ± 48.9	1106 ± 45	47.5 ± 12.3	47.7 ± 10.0
Chamling et al. [15]	1 year	No therapy	1.5 T	septal	1096 (1049–1128)	1095 (1078–1123)	57 (48–66)	60 (51–69)
		Tafamidis 61 mg		septal	1111 (1094–1125)	1111 (1088–1131)	64 (52–70)	63 (54–68)
Takashio et al. [16]	1 year	Tafamidis	N/A	N/A	1414 ± 54	1421 ± 64	52.2 ± 11.2	53.2 ± 11.7
Tsai et al. [21]	1 year	Tafamidis 61 mg	1.5 T	septal	1159.5 ± 53.0	1157.4 ± 68.2	51.5 ± 8.9	49.0 ± 9.4
Ney et al. [22]	6 months	Tafamidis 61 mg	1.5 T	global	1095 (1026–1119)	1074 (1045–1100)	46 (41–51)	47 (43–55)

ECV, extracellular volume fraction, N/A, not applicable. Values are mean ± standard deviation or median (inter quartile range).

## Data Availability

The data that support the findings of this study are available from the corresponding author, [S.K.], upon reasonable request.

## Supplementary Materials

The following supporting information can be downloaded at: https://www.mdpi.com/article/10.3390/tomography10080097/s1, Supplemental File S1. Database search. Table S1. Newcastle—Ottawa quality assessment scale case control studies. Figure S1. Funnel plot of Baseline ECVs before tafamidis treatment. Figure S2. Funnel plot of Baseline ECVs after tafamidis treatment.
