# Supplementary material for: Monitoring the Efficacy of Tafamidis in ATTR Cardiac Amyloidosis by MRI-ECV: A Systematic Review and Meta-Analysis
Source: Tomography. 2024 Aug 16;10(8):1303–11. doi: 10.3390/tomography10080097 (PMC11360159; doi:10.3390/tomography10080097)
Supplement: Supplementary file 1 [file tomography-10-00097-s001.zip › tomography-3052855-supplementary.pdf]

Supplemental material S1

#1 "transthyretin amyloid cardiomyopathy" OR "ATTR-CM" OR "ATTR cardiomyopathy"

#2 "extracellular volume" OR "ECV" OR "MRI"

#3 "tafamidis" OR "tafamidis treatment" OR "Monitoring"

#4 #1 AND #2 AND #3

Number of articles

-Pubmed 135

-WOS 185

-Embase 310

-Cochrane library 62

Supplemental material S2

NEWCASTLE - OTTAWA QUALITY ASSESSMENT SCALE CASE CONTROL STUDIES

**Study**	**Selection (Max=4)**	**Comparability (Max=2)**	**Exposure (Max=3)**	**Total Score**
Fontana et al.	4	2	3	9
Rettel et al.	4	2	3	8
Chamling et al.	4	1	3	8
Takashio et al.	4	2	3	9
Tsai et al.	4	2	3	9
Ney et al.	4	2	3	9

Supplemental material S3

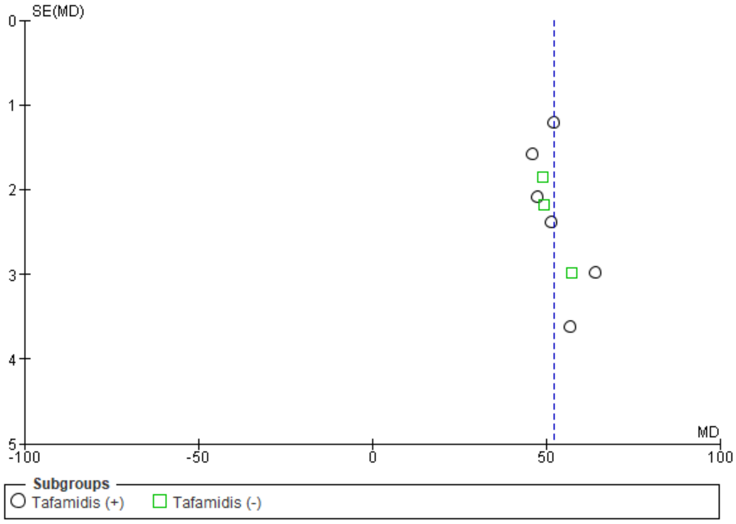

Funnel plot of Baseline ECVs before tafamidis treatment

Begg's test yielded the following results.

Rank Correlation Test for Funnel Plot Asymmetry

Kendall's tau = 0.5353, p = 0.0464

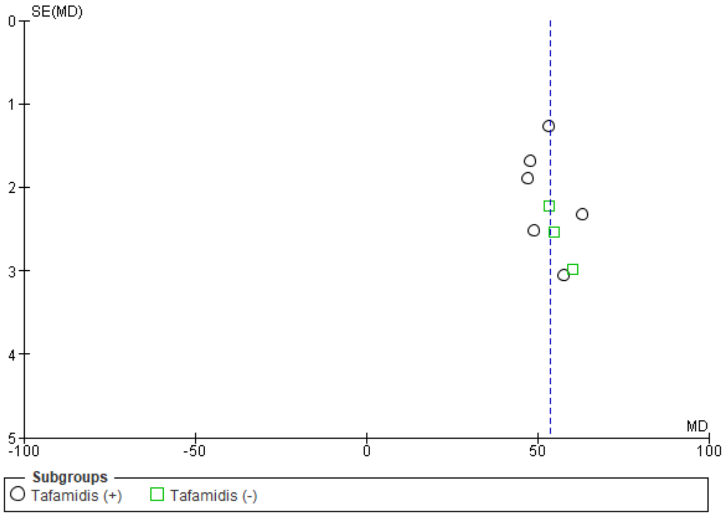

Funnel plot of Baseline ECVs after tafamidis treatment

Begg's test yielded the following results.

Rank Correlation Test for Funnel Plot Asymmetry

Kendall's tau = 0.3889, p = 0.1802
